# Sequence periodicity of *Escherichia coli *is concentrated in intergenic regions

**DOI:** 10.1186/1471-2199-5-14

**Published:** 2004-08-26

**Authors:** Sergey Hosid, Edward N Trifonov, Alexander Bolshoy

**Affiliations:** 1 Genome Diversity Center, Institute of Evolution, University of Haifa, Mt. Carmel 31905 ISRAEL

## Abstract

**Background:**

Sequence periodicity with a period close to the DNA helical repeat is a very basic genomic property. This genomic feature was demonstrated for many prokaryotic genomes. The *Escherichia coli *sequences display the period close to 11 base pairs.

**Results:**

Here we demonstrate that practically only ApA/TpT dinucleotides contribute to overall dinucleotide periodicity in *Escherichia coli*. The noncoding sequences reveal this periodicity much more prominently compared to protein-coding sequences. The sequence periodicity of ApC/GpT, ApT and GpC dinucleotides along the *Escherichia coli *K-12 is found to be located as well mainly within the intergenic regions.

**Conclusions:**

The observed concentration of the dinucleotide sequence periodicity in the intergenic regions of *E. coli *suggests that the periodicity is a typical property of prokaryotic intergenic regions. We suppose that this preferential distribution of dinucleotide periodicity serves many biological functions; first of all, the regulation of transcription.

## Background

DNA sequence periodicity with the period about 10–11 base pairs (bp) has been long known in eukaryotic DNA sequences. It was discovered recently in prokaryotic sequences as well [1-6]. The periodicity in Eubacteria sequences usually shows the period close to 11 bp [1]. This period is clearly different from the structural helical period of 10.5–10.6 bp/turn [7,8]. The difference was interpreted [1,2] as a possible reflection of the sequence dependent writhe of prokaryotic DNA. In the work [9] it was demonstrated that the periodicity in the bacterial genomes, in *E. coli *as well, is distributed in a non-uniform way, in scattered segments of the size 100–150 bases. It was also known for a long time that quite a few DNA promoter regions of *E. coli *possess the sequence periodicity of AA and TT dinucleotides [10].

The sequence periodicity of AA/TT dinucleotides is frequently associated with sequence-dependent DNA curvature, which is known to play an important role in the initiation of transcription of many genes (for reviews, see [11-15]). Using different models and approaches for prediction of intrinsic DNA curvature it was shown that many *E. coli *promoters have upstream curved sequences [16,17]. Pedersen et al. [18] showed that promoter area frequently has an unusual sequence structure. This region possesses higher DNA curvature, more rigid and less stable. Moreover, in our study of prokaryotic terminators of transcription (Hosid and Bolshoy, submitted) we have found that in *E. coli *DNA curvature peaks are frequently located downstream of the CDS.

Since the dinucleotide periodicity with the period close to the helical repeat is associated with DNA intrinsic curvature [19-23], the curvature distribution along DNA would suggest similar distribution of DNA sequence periodicity.

In this work, the sequence dinucleotide periodicity in *E. coli *and its distribution along the genome are systematically analyzed. A strong preference of intergenic regions to express the sequence periodicity of AA, AC, GC, and TT dinucleotides is discovered.

## Results and Discussion

**Figure 1:**
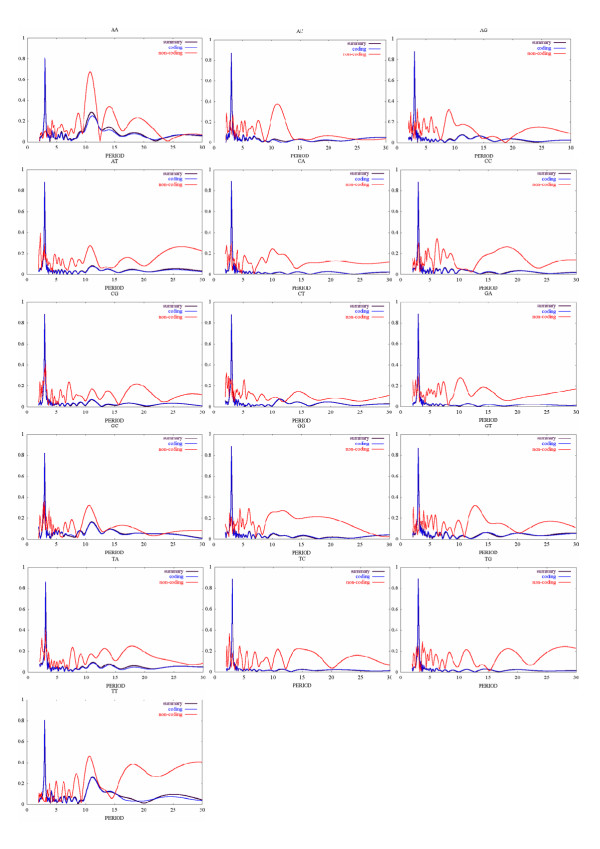
Periodograms of the distance distributions of 16 dinucleotides in *E. coli *genome. The complete nucleotide sequence of *E. coli, *as well as subsets of its coding and noncoding regions, was subjected to the positional autocorrelation analysis for all 16 dinucleotides separately. Resulting autocorrelation profiles were after that analyzed by Fourier transform. The black lines correspond to the whole genome, the blue curves – to the coding sequences, and the red curves – to the noncoding sequences.

Positional autocorrelation analysis of the nucleotide sequences is an appropriate tool to detect all major characteristic distances in the sequences, the periodicities in particular. The complete genome of *E. coli, *as well as its coding and noncoding regions, was subjected to this procedure. Resulting autocorrelation profiles for all 16 dinucleotides (data not shown) were further analyzed by Fourier transform. In Fig. 1 the corresponding spectra are shown. The analysis demonstrates presence of the sequence periodicity of AA and TT dinucleotides with a period close to 11 bp mostly in intergenic regions, and weaker periodicity of AC and GC notably exclusively in intergenic regions. All 16 dinucleotides show periodicity of 3 bp, a well-known characteristics of the coding sequences, e.g. [24,25]. Weak 2 bp periodicity of AT and TA is also observed in intergenic regions. It indicates, perhaps, presence of tandem ApT repeats. A weak 10 bp periodicity of GC in intergenic regions, probably, corresponds to terminator regions (work in progress). The amplitudes of the 11 bp periodicity of AA and TT are the highest, even comparable with 3 bp coding periodicity. We, thus, focused on AA and TT distributions.

To screen the genome of *E. coli *and find out where the periodical regions are located, we chose the period 11.2 bp [1,2,5] and this study (Fig. 1); and the window of 150 bp [9,26]. We used periodical AA and TT probes with the above periodicity to correlate with the *E. coli *genome sequence and to detect the periodical sites. This calculation shows that the periodicity is not evenly distributed along the *E. coli *genome.

**Figure 2:**
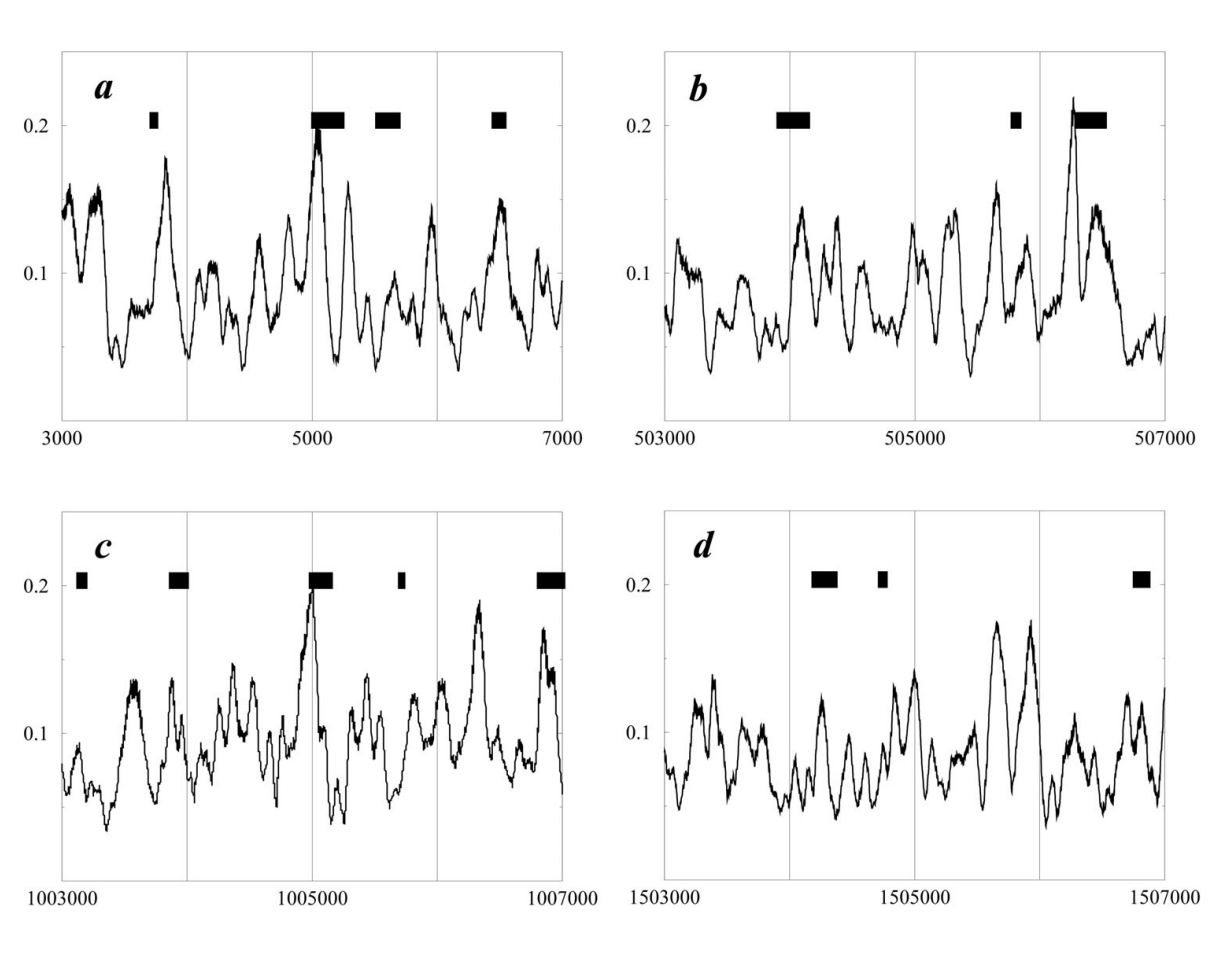
Four examples of periodicity maps for fragments of *E. coli *genome. The maps were smoothed by running average with window 51 bp. The black bars on the top of the plot correspond to positions of intergenic regions.

In Fig. 2, the typical maps for several large segments of the *E. coli *genome are shown. The periodicity is distinctly located in certain regions. Many of the peaks observed are found to correspond to the intergenic regions (indicated by the black bars at the top). For example, two such peaks of periodicity in Fig. 2a correspond to the intergenic regions. Three such maxima are observed in Fig. 2b, three in Fig. 2c, and two in Fig. 2d. For the genome sections in Fig. 2 about 2/3 of the intergenic regions are associated with the local periodicity.

**Figure 3:**
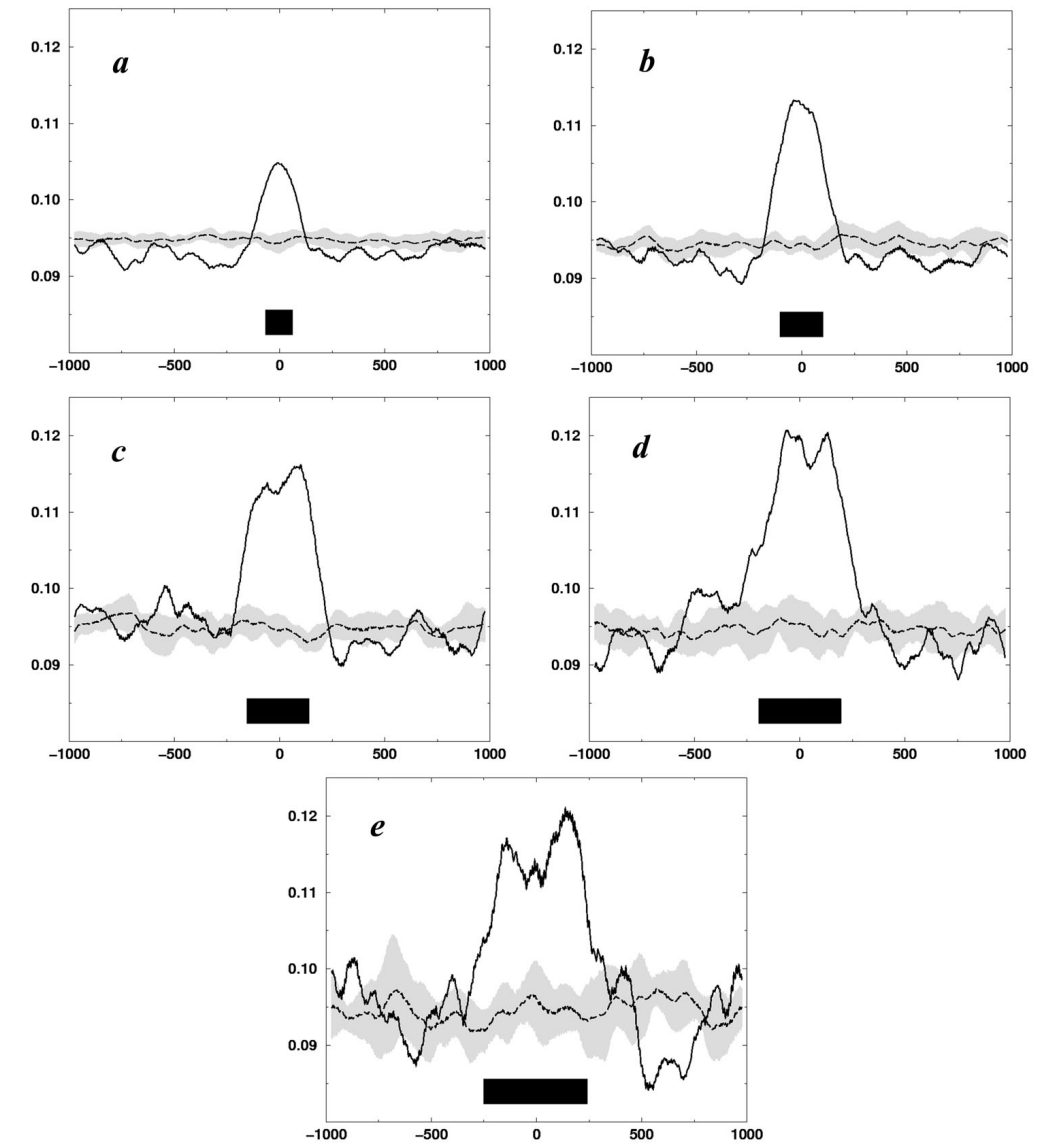
The averaged maps of periodicity are synchronized at the centers of intergenic regions and smoothed by a running average of 51 bp. Five families of the intergenic regions with different lengths are presented: a) 100 ± 50, bp 1073 sequences, b) 200 ± 50 bp, 602 sequences, c) 300 ± 50 bp, 319 sequences, d) 400 ± 50 bp, 160 sequences, and e) 500 ± 50 bp, 78 sequences. The black bars at the bottom of the each figure correspond to the average intergenic region. The gray bands around black dashed lines correspond to standard deviations around randomized background.

To verify the apparent strong correlation between the intergenic regions and AA/TT periodicity, we split intergenic regions in several families by size and analyzed the subsets separately by aligning (centering) the regions and summing up the respective local periodicity distributions. The combined maps for intergenic regions with a size from 50 to 150 bp, from 150 to 250 bp, from 250 to 350 bp, from 350 to 450 bp, and from 450 to 550 bp are shown in Fig. 3. This figure demonstrates, indeed, that intergenic regions are typically periodic, irrespective of the size. The average amplitudes of the observed periodicities – 0.1–0.25 units – are comparable with the amplitudes in Fig. 2, which indicates that, indeed a large proportion of the intergenic regions are periodical.

**Figure 4:**
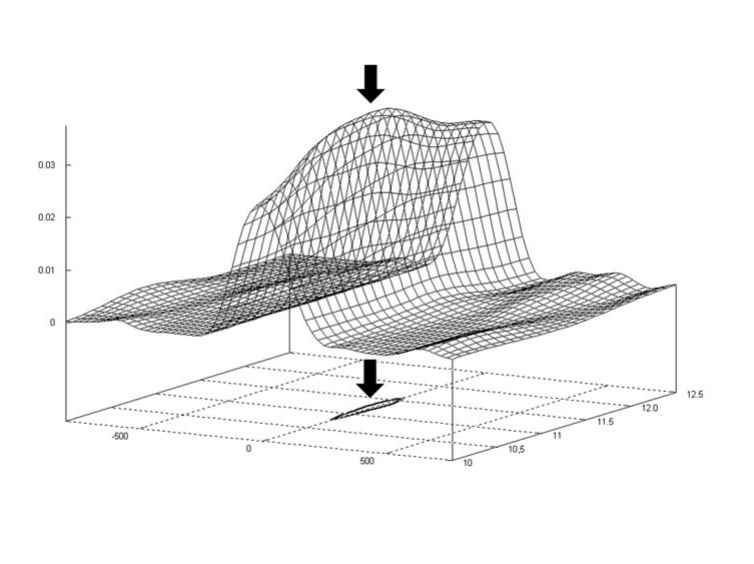
The 3D resonance plot for the intergenic regions of length 200 ± 50 bp. The maximum of resonance the plot corresponds to period 11.3 ± 0.4 bp. The contour around the maximum is also shown as a projection at the base line level.

To verify the choice of the period 11.2 bases, we calculated the periodicity maps for highly populated group of the regions of the size 200 ± 50 bp, by assuming different periods in the range 10.5–12.5 bases. The resonance 3D plot in Fig. 4 indicates that the best-fit period is 11.3 ± 0.4 bp, which confirms earlier estimates of the *E. coli *DNA sequence periodicity.

The spectral analysis (Fig. 1) and examples of the periodicity distribution maps (Fig. 2) show that apart from described correlation among the intergenic regions and AA/TT periodicity, there are numerous sites of periodicity located within coding sequences. Work is in progress to find out the functional relevance, if any, of these sites.

## Conclusions

The observed concentration of the sequence periodicity in the intergenic regions corroborates earlier results and suggests that the periodicity is a typical property of the intergenic regions.

## Methods

### Genome data

The sequence of the whole genome of *Escherichia coli *K-12 MG1655, locus U00096, 4639221 base pairs, was taken from the National Center of Biotechnology Information . Intergenic regions were identified in accordance with the annotation to this genome of *E. coli *and gathered in a separate dataset.

### Fourier transform of positional autocorrelation function

Autocorrelation profile *X *was calculated for each dinucleotide separately. For the calculation of ApA autocorrelation, for example, we calculated the number of occurrences of pairs ApA – ApA in a distance *k*, and designated it by *X*_*k*_. Spectral analysis of autocorrelation profile *X *was obtained using the following formulae:



where *f*_*p *_is normalized wave-function amplitude of period *p*, *X *is an autocorrelation profile for one chosen dinucleotide, *X*_*i *_is its value in position *i*,  is its average value, and *W *is a maximal considered autocorrelation distance (in our case 100 bp).

### Sequence periodicity

As a probe of periodicity the sine waves with period *T *were taken to describe idealized periodical distribution of AA and TT dinucleotides within window *W*. The probes were correlated with *E. coli *sequences by moving the probes along the sequences and calculating the value *C *for every position.



where *i *is an index of a dinucleotide position in the window *W *and



The value *C*_max _is introduced for the normalization purposes. It is calculated as follows:



where *i *is a position in the window *W *and



Ideally periodical sequence segments would be, therefore, described by *C *= 1, while segments with no periodicity would correspond to *C *= 0. The results of these calculations are presented as maps of the sequence periodicity. The four sample maps are shown in Fig. 2a,2b,2c,2d.

### Synchronization of the maps

The maps around intergenic regions were combined (summed) separately for the groups of similar sizes of the intergenic regions. Five such groups were analyzed: 100 ± 50 bp, 200 ± 50 bp, 300 ± 50 bp, 400 ± 50 bp, and 500 ± 50 bp. For each group the maps were synchronized at the respective intergenic centers and the sums of the maps were calculated and smoothed by a running average within 51 bp. The standard deviations for the combined plots were estimated by generating random sequences of the same size and dinucleotides composition for each group separately and averaging the respective periodicity maps.

### The resonance plot

The resonance 3D plot for the intergenic regions of length 200 ± 50 bp was built from calculations with different periods *T *in the interval 10–12.5 bp. One-third (202) of the most periodic maps of this group was taken for the calculation. The maps for different periods *T *were smoothed five times by a running average over 51 bp. The baselines were set to 0. The surface of 3D plot was smoothed 3 times by a running average over 9 point square elements, on the grid with separations 0.1 bp for *T*, and 20 bp for sequence position.

## Competing interests

None declared.

## Authors' contributions

SH carried out all graphics. ENT and AB participated in the design of the study and analysis of results. All authors drafted the manuscript. All authors read and approved the final manuscript.
